# Outcome Assessment of the Marshall Coughing Test during Cervix Reposition Maneuver in Women with Urinary Stress Incontinence with/without Genital Prolapse

**DOI:** 10.5402/2012/109858

**Published:** 2012-02-20

**Authors:** Vesna Antovska

**Affiliations:** Department for Urogynaecology and Pelvic Floor Disorders, University Clinic for Gynaecology and Obstetrics, Medical Faculty, University “Saint Cyril and Methodius”, Vodnjanska 17, 1000 Skopje, Macedonia

## Abstract

*Objectives*. Outcome assessment of the Marshall coughing test (MT) during cervix reposition maneuver (CRM) in women with urinary stress incontinence (USI) with/without genital prolapse (GP). *Study Design*. 268 patients, divided into USIg (*n* = 132) with isolated USI and USIGPg (*n* = 136) with USI and GP stage I/II, additionally divided into USIGP(A) (*n* = 78) with USI and GP stage I and USIGP(B) (*n* = 58) with USI and GP stage II, were evaluated with pelvic organ prolapse quantification (POPQ), MT, and CRM. *Results*. (a) 7.58% had (+) MT with CRM in USIg; (b) in up to 96.15% MT became negative during CRM in USIGP(A); (c) in 51.72% MT became positive only during CRM, as a sign for occult USI in USIGP(B); (d) point Aa (POPQ), which is bladder neck(BN) projection on the anterior vaginal wall, was situated higher in rest position (RP), but moved lower during the Valsalva maneuver (VM) in USIg versus USIGPg (*P* < 0.05). *Conclusion*. CRM could be useful arm in selection of (1) patients with isolated USI and great chance for postoperative failure; (2) patients with USI+GP stage I, who need GP repair during antistress surgery; (3) patients with USI + GP stage II, who need antistress procedure during vaginal hysterectomy.

## 1. Introduction

Women with genital prolapse (GP) may be continent paradoxically, as a result of urethral kinking or compression, but after repair of GP, 22–80% of patients will present de novo urinary stress incontinence (USI) [1]. These women have occult USI and can be preoperatively identified by performing the barrier test, which is actually a stress test to determine whether urine leakage occurs during GP-reposition. This test can be performed in several different ways, such as using a pessary, vaginal pack, or Sims' speculum or performing the cervix reposition maneuver (CRM), which is simple imitation of postoperative pelvic organ position with grasping the cervix with tenaculum and pushing it in upwards/backwards direction to the promontorium. The barrier test can also be performed during urodynamic investigation in sitting position with the bladder at maximum cystometric capacity, because if it is performed at a lower bladder volume, the prolapse may still mask USI. Klutke and Ramos [2] suggested that a negative reposition test is reliable in the prediction of patient who will be stress continent after prolapse repair. Gordon et al. [3] reported that 50% of clinically continent women with severe pelvic organ prolapse, who had a preoperative positive barrier test, experienced de novo USI after prolapse repair. Lecuru et al. [4] in their series of 203 abdominal correction of GP reported 86.7 to 100% anatomically good long-term results, but only 53.3 to 80.5% functionally good. In a study of 191 patients with GP [5], in 50% this prolapse was combined with USI. Vaginal prolapse recurred after 4 and 12 months in 4 and 6% of cases, respectively, but up to 31% still complained of USI 4 months after the operation. Morley and DeLancey [6] in 57 patients with sacrospinous suspension had 12.28% recurrent vaginal prolapse and 15.79% postoperative USI. Hewson [7] had only 60% cure of USI with additional buttressing sutures and only 35% improvement of intercourse. Maher et al. [8], in order to determine the effects of many different surgeries used in the management of pelvic organ prolapse, searched the Cochrane Incontinence Group Specialised Register (9 February 2009) and reference lists of relevant articles. According to their analysis of 40 randomized controlled trials which included 3773 women, the continence surgery at the time of prolapse surgery in continent women did not significantly reduce the rate of postoperative stress urinary incontinence (RR 1.39, 95% CI 0.53 to 3.70; random-effects model).

The central question in the preoperative evaluation of women with USI in presence or absence of GP is to estimate functional outcome once the anatomy is corrected. It is well documented that correction of the anatomy can decrease resistance to the urethra, thereby unmasking intrinsic sphincter deficiency [9]. On the other hand, the different antistress procedures in patients with USI without GP also can result in postoperative failure, perhaps due to vertical pelvic organ position. Different authors report different failure rate, from more than 30% to less than 10% [10–12]. Nevertheless, the cure rate shows a decreasing trend over the time. So, within the first year of treatment, the overall continence rate is approximately 85–90%, but after 5 years, approximately 70% of patients can expect to be dry [13]. According to the integral theory of continence of Ulmsten, Petros, and Woodman [14, 15], the forward muscle force of m. pubococcygeus stretches the vagina forwards against the pubourethral ligament to close the urethra behind, and backward forces stretch the upper vagina and bladder base backwards and downwards in a plane around the pubourethral ligament to close off the proximal urethra. In patients with vertical pelvic organ position, perhaps this mechanism does not work quite effectively.

## 2. Material and Methods

### 2.1. Eligibility Criteria for Participants

Presence of USI with or without GP stage I or II (according to the POPQ system) [16].

### 2.2. The Setting, Location, and Timing: Where and When the Data Were Collected

The Department for Urogynecology and Pelvic Floor Disorders in the Clinic of Gynecology and Obstetrics, Medical Faculty of the “Saint Cyril and Methodius” University, in Skopje in the period from the 1st of January 2010 to the 31st of December 2010. The study was designed according to the CONSORT statement [17].

### 2.3. Precise Details of the Interventions for Each Group: How and When They Were Actually Administered

The experimental arms are (1) coexisting presence of GP stage I/II and USI, that is, USIGP group (USIGPg) (*n* = 136) and (2) presence of USI without GP, that is, USI group (USIg) (*n* = 132). The study was approved by the local research ethics committee of the Macedonian Association of Gynecologists and Obstetricians. The clinical assessment of patients was performed by the author of this paper. The study was a prospective observational study with no allowance for patient preference.

### 2.4. How Sample Size Was Determined and the Method Used to Generate the Random Allocation Sequence, Including Details of Any Restriction

Every postmenopausal patient with isolated USI, or with USI and GP stage I/II, admitted to our Department in the above-mentioned period, was assessed for eligibility (*n* = 278). Ten patients were excluded from the study because they were unwilling to participate. So, 268 patients were randomised, and all of them completed the study. After they underwent the complete urogynecologic examination according to our Urogynecologic Protocol, they were divided into two groups: USIGPg (*n* = 136) with GP and USI and USIg (*n* = 132) only with USI. Additionally, USIGPg was divided into two subgroups: USIGP(A) (*n* = 78) with GP stage I and USIGP(B) (*n* = 58) with GP stage II. All participants were aware of group assignment. All subjects were given an explanation of the study, and written informed consent was obtained.

### 2.5. Specific Objectives and Hypotheses

The purpose of the study was an outcome assessment of the Marshall coughing test (MT) during cervix reposition maneuver (CRM) in lithotomic position in patients with USI with/without GP. The hypotheses were as follows: (1) about 10% of patients with isolated USI have positive MT only in upright position, (2) about 10% of patients with isolated USI have positive MT during CRM; (3) more than 80% of patients with manifest USI and GP stage I have negative MT during CRM; (4) about 20% of patients with USI and GP stage II have positive MT during CRM, as a sign of occult USI.

### 2.6. Clearly Defined Primary and Secondary Outcome Measures: All Methods Used to Enhance the Quality of Measurements

1. *Demographic data*, such as age, duration of postmenopausal age, parity, BMI, and occupation, were evaluated.
2. C*omplete evaluation for urinary incontinence: *a structured questionnaire for urinary symptoms based on the International Continence Society recommendation [18]; multichannel urodynamic examination: retrograde provocative multichannel urethrocystometry, passive and dynamic urethral pressure profilometry, cough and the Valsalva leak point pressure, and simple uroflowmetry with postvoid residual urine volume; MT in upright and lithotomic position, as well as lithotomic position with artificial cervix reposition that is, cervix reposition, maneuver (CRM) for detection of occult USI, after bladder filling with 300 mL 3% boric acid.
3. *Complete evaluation for GP*: a structured questionnaire and pelvic organ prolapse quantification (POPQ) during pelvic examination performed in the supine position in a birthing chair in rest position (RP) and while performing the Valsalva maneuver (VM) with maximal effort. The bladder was empty by catheterization and rectum too, by morning defecation.

### 2.7. Statistical Methods

The Student's paired test was used for comparing the demographic data and POPQ and its anatomic landmarks. Mantel-Haenszel's *χ* ^2^ test was used for comparing the demographic data and urodynamic parameters according to the following formula:

(1) $$\chi^{2}\;=\frac{n\;{\left(\left[AD-BC\right]-\left(n/2\right)\right)}^{2}}{\left(A+B\right)\left(C+D\right)\left(A+C\right)\left(B+D\right)}.$$

## 3. Results

There were some significant differences in demographic data. So, USIGPg had longer duration of postmenopausal age (*P* < 0.01). Regarding the occupation in USIGPg farmers and retired persons dominated (*P* < 0.05; *P* < 0.001, resp.), but in USIg there were more factory workers and clerk/teachers (*P* < 0.05; *P* < 0.001, resp.) (Table 1).

Regarding POPQ and its anatomic landmarks, there were differences between the groups, but also into the same group during VM versus RP (Table 2): (1) point Aa, which is bladder neck (BN) projection on the anterior vaginal wall, was situated higher in RP, but moved significantly lower during VM in USIg versus USIGPg (*P* < 0.05); (2) point C, which represents the leading edge of cervix, was situated significantly lower in RP, but especially during VM in USIGPg versus USIg (*P* < 0.001; *P* < 0.001, resp.); (3) the total vaginal length (tvl) was significantly greater in USIg versus USIGPg in RP (*P* < 0.05).

We analyzed the results of MT into the groups. In USIg, we found the following: (1) positive MT only in upright position in 15.15% of cases; (2) positive MT in lithotomic and upright position in 77.27% of cases; (3) positive MT in all three positions in only 7.58% of patients; (4) in up to 92.24% of patients, MT became negative during CRM, as a prognostic sign for good postoperative results. In USIGP(A), we found the following: (1) positive MT in lithotomic and upright position in 89.74% of cases; (2) positive MT in all three positions in 3.85% of cases; (3) positive MT only in upright position in only 6.41% of patients; (4) up to in 96.15% of patients, MT became negative during CRM, as a prognostic sign for good postoperative results. In USIGP(B) we found the following: (1) positive MT in lithotomic and upright position in 34.48% of cases; (2) positive MT only in lithotomic position in 13.79% of cases; (3) up to in 51.72% of patients, MT became positive only during CRM, as a prognostic sign for poor postoperative results after the correction of GP that is, for great possibility of de novo USI (Table 3).

In Table 4 we represent the differences among the groups regarding the presence of USI during the three positions: (1) USI was more frequent in USIg and USIGP(A) versus USIGP(B) in lithotomic position (*P* < 0.001; *P* < 0.001, resp.) and in upright position (*P* < 0.001; *P* < 0.001, resp.); (2) USI was more frequent in USIGP(B) versus USIg and USIGP(A) during CRM (*P* < 0.001; *P* < 0.001, resp.).

In Table 5 we represent the differences among the groups regarding the division of patients with different combinations of MT in the three positions. So (1) positive MT in all three positions was more frequent in USIg versus USIGP(B) (*P* < 0.05); (2) positive MT only in upright position (perhaps due to vertical pelvic organ position) was more frequent in USIg versus USIGP(B) (*P* < 0.01); (3) positive MT only in lithotomic position was significantly less frequent in USIg versus USIGP(A) and USIGP(B) (*P* < 0.001; *P* < 0.001, resp.); (3) negative MT only with CRM in favor of excellent chances for postoperative success was more frequent in USIGP(A) versus USIg (*P* < 0.01), but also less frequent in USIGP(B) versus USIg and USIGP(A) (*P* < 0.001; *P* < 0.001, resp.). These findings were in favor of USIGP(A) having the best postoperative results and USIGP(B) having the worst ones; (4) positive MT only with CRM, as a sign of occult USI, was more frequent in USIGP(B) versus USIg and USIGP(A) (*P* < 0.001; *P* < 0.001, resp.).

In Table 6 we represent the differences in frequency of the functional symptoms according to the questionnaire/clinical examination between USIGPg and USIg. So (1) occult USI, which was evident only during CRM, predominated in USIGPg (*P* < 0.001), but genuine USI in USIg (*P* < 0.001). The symptoms, such as incomplete emptying and weak stream, were also predominant in USIGPg (*P* < 0.001; *P* < 0.001, resp.); (2) bowel symptoms, such as flatus incontinence, urgency of defecation, discomfort with defecation, constipation, digital manipulation to finish defecation, and feeling of incomplete evacuation predominated in USIGPg (all with *P* < 0.001); (3) sexual symptoms, such as: painful coitus, unsatisfactory coitus, decrease in orgasmic response, and incontinence during intercourse predominated in USIGPg (all with *P* < 0.001); (4) other local symptoms, such as vaginal pressure and heaviness, awareness of tissue protrusion, low back pain, abdominal pressure, and observation or palpation of a mass, also were predominant in USIGPg (all with *P* < 0.001).

Comparing the urodynamic analyses in both groups (Table 7), we found that positive dUPP, as a urodynamic evidence of USI, was significantly predominant in USIg (*P* < 0.001), but negative dUPP, as a urodynamic evidence of absence of USI, was significantly predominant in USIGPg (*P* < 0.001). The other urodynamic parameters did not show significant differences between the groups. The urodynamic analyses in USIGPg were performed without any pessary in situ.

## 4. Discussion

(1) In USIg with present USI without GP, we noticed positive MT only in upright position in 15.15% of cases. According to the integral theory of continence of Ulmsten, Petros, and Woodman [14, 15], the backward forces stretch the upper vagina and bladder base backwards and downwards in a plane around the pubourethral ligament to close off the proximal urethra. We think that in patients who have positive MT only in upright position, the pelvic organs are placed in more vertical position than usual. This change in pelvic organ position perhaps is due to the changes of pelvic skeleton, such as upwards and downwards rotation of the promontorium and excessive lumbarlordosis. In this situation the backwards forces would not be enough strong to ensure a proper function of the urethra-closure mechanism that is, this mechanism does not work quite effectively. To improve the cure rate of antistress procedures, in these cases we recommend a complementary performance of procedure which would supply a horizontalisation of the vagina, for example, Young's plication of the uterosacral ligaments or sacrocolpopexy. The noted percentage of positive MT only during upright position in patients with isolated USI almost confirmed our first hypothesis.

In the same group of patients we noticed that in 7.58% of cases, the Marshall test was positive during CRM. We value this finding as very convincing sign of future anti-stress surgery failure. Therefore, we recommend CRM as an obligatory part of preoperative evaluation in order to predict the postoperative failure. In presence of positive MT with CRM, a clear explanation should be given to the patient for avoiding an unpleasant postoperative surprise. As a confirmation of these, our statement is the fact that this percent, which we found as positive MT during CRP, is similar to those reported by most authors as a failure rate after anti-stress surgery [10, 19]. The noted percentage of positive MT during CRM in patients with isolated USI gave an affirmation of our second hypothesis.

(2) In USIGP(A)s with USI and GP stage I, in up to 96.15 % (75/78), MT became negative during CRM. According to our results, in all patients with USI and GP stage I in whom positive MT became negative during CRM, we recommend a concomitant performance of an anti-stress procedure, abdominal hysterectomy, and a procedure which would provide a stable position of the vagina in high level, such as Young's plication of the uterosacral ligaments or sacrocolpopexy. An isolated anti-stress procedure would have short-term results, that is, an early appearance of recurrent USI and vault prolapse would happen. In these patients, the deteriorating process of pubourethral ligaments due to downwards forces produced by the uterine prolapse is already finished, but the cystocele is not large enough to mask USI in lithotomic and upright position. An isolated anti-stress procedure will not solve the problem, that is, stop this deteriorating process because the downwards GP forces will continue and soon will eliminate the positive postoperative results, resulting in recurrent USI and vault prolapse. Therefore, we recommend obligatory GP repair during anti-stress surgery in order to obtain a durable postoperative success. Costantini et al. [20], in their study of 47 women with GP and USI, divided into two groups: group A, treated with GP repair and Burch colposuspension and group B, treated only with GP repair, did not find significant intergroup difference regarding anatomical outcome, but significant intergroup differences regarding recurrent USI (in group A 13/24 patients were still incontinent compared with 9/23 in group B).

The noted percentage of negative MT during CRM in patients with USI and GP stage I of our study slightly exceeded our expectation in the third hypothesis.

(3) In USIGP(B) with USI and GP stage II, we found that up to in more than half of patients, the Marshall test became positive only during CRM. This maneuver corrected the cystocele and annulated its immobilizing effect on the bladder neck, that is, allowed a complete movement downward and backward of the bladder neck and its opening during VM with consecutive USI development. The noted percentage of positive MT during CRM in patients with USI and GP stage II in our study considerably exceeded our expectation in the forth hypothesis. This is the reason why we strongly recommend CRM in preoperative evaluation of patients with stage II GP. In cases with USI and GP stage II, we always combined the vaginal hysterectomy with the anti-stress procedure: Lazarevski's suburethral vaginal duplication [21]. In these group of patients, the deteriorating process of pubourethral ligaments, due to downward GP forces, is already finished, but the cystocele is large enough to have an overall impact over the above-mentioned forces and mask USI in lithotomic/upright position. Therefore, we recommend CRM in preoperative evaluation and some anti-stress procedure during vaginal hysterectomy, in order to avoid postoperative de novo USI.

Liang et al. [22] in their study of 79 patients with severe GP, diagnosed 49/79 (62.03%) as of the occult USi with preoperative performing the pessary test. In 32 of them, they performed TVT during vaginal hysterectomy with cure rate of USI of 90.6%. In the other 17 women with occult USI, they performed only vaginal hysterectomy and noted 64.7% postoperative de novo USI. The patients with negative preoperative pessary test did not develop de novo USI. In another study done by us of 216 patients with genital prolapse: stage III/IV (POPQ), we diagnosed occult USI with CRM in 31/216 (14.4%) and genuine USI in 12/216 (5.56%). In all cases we systematically performed the Lazarevski's vaginal duplication as an antistress procedure during vaginal hysterectomy. At the last follow-up (mean 38.6 months), we noted only 3 recurrent USI of 31 cases with preoperative occult USI, that is, 90.32% cure rate, but also 3 recurrent USI of 12 patients with preoperative genuine USI, that is, 75% cure rate unpublished observations. Chaikin et al. [23], in 14 patients with GP and occult USI, performed pubovaginal sling and noted 14% of postsurgical USI development.

Cervix reposition maneuver could be useful arm in selection of (1) patients with isolated USI and great chance for postoperative failure; (2) patients with USI and GP stage I, who need GP repair during anti-stress surgery; (3) patients with USI and GP stage II, who need anti-stress procedure during vaginal hysterectomy.

## Figures and Tables

**Table 1:** Demographic data: age, duration of the postmenopausal age, parity, body mass index (BMI), and ocupation in USIGPg (*n* = 136) and USIg (*n* = 132).

Variable	USIGPg (*n* = 136)	USIg (*n* = 132)	*t*/*χ* ^2^
Age (years) (mean ± SD) 1	61.3 ± 4.5	52.2 ± 5.2	1.32
Duration of postmenopausal period (years) (mean ± SD) 1	13.4 ± 3.3	3.7 ± 3.1	**2.96** ^(∗)^
Parity (mean ± SD)1	3.2 ± 0.9	2.1 ± 0.6	0.60
Body mass index (mean ± SD)1	28.3 ± 4.1	34.3 ± 4.2	1.02
Factory worker 2	17/136	29/136	6.33^(†)^
Farmer 2	42/136	25/132	4.49^(†)^
Clerk/teacher 2	8/136	28/132	14.93^(‡)^
Housewife 2	25/136	35/132	3.05
Retired person 2	44/136	15/132	16.03^(‡)^

(1) Student's paired test: ^(†)^ *P* < 0.05; ^  (∗)^ *P* < 0.01; ^(‡)^ *P* < 0.001. (2) Mantel-Haenzel's *χ* ^2^ test with df of 1: ^(†)^ *P* < 0.05; ^  (∗)^ *P* < 0.01; ^(‡)^ *P* < 0.001.

**Table 2:** Quantitative description of pelvic organ position (POPQ) with anatomic landmarks in USIGPg (*n* = 136) and USIg (*n* = 132).

POPQ	Rest position		Valsalvas maneuver		*t*_1_	*t*_2_	*t*_3_	*t*_4_
	USIGPg (*n* = 136)	USIg (*n* = 132)	USIGPg (*n* = 136)	USIg (*n* = 132)				
	(1)	(2)	(3)	(4)	1-2	3-4	1–3	2–4
Aa (cm)	−1.23 ± 0.66	−1.56 ± 0.78	−2.39 ± 0.68	−0.80 ± 0.44	0.32	**1.97** ^†^	1.23	0.85
Ba (cm)	−0.99 ± 0.88	−1.43 ± 0.57	+0.89 ± 0.84	−0.33 ± 0.24	0.50	1.39	0.55	1.79
C (cm)	−1.56 ± 1.11	−7.24 ± 0.25	+0.55 ± 1.23	−5.12 ± 0.88	**5.00** ^‡^	**3.75** ^‡^	1.27	2.53*
D (cm)	−4.83 ± 1.36	−7.55 ± 0.77	−4.19 ± 1.22	−5.86 ± 0.99	1.74	1.06	0.35	1.35
Bp (cm)	−2.22 ± 0.45	−2.75 ± 0.55	−1.10 ± 0.97	−2.48 ± 0.66	0.75	1.18	1.05	0.31
Ap (cm)	−2.00 ± 0.99	−2.75 ± 0.55	−1.10 ± 0.97	−2.48 ± 0.66	0.66	0.18	0.65	0.31
gh (cm)	4.67 ± 0.95	3.74 ± 0.99	5.11 ± 0.69	4.11 ± 0.89	0.68	0.89	0.38	0.28
tvl (cm)	4.51 ± 1.12	7.33 ± 0.55	3.84 ± 0.87	5.58 ± 0.68	2.27*	1.58	0.47	1.76
pb (cm)	2.18 ± 0.46	2.67 ± 0.52	2.16 ± 0.77	2.45 ± 0.78	0.71	0.27	0.02	0.23

Student's paired test: ^(†)^ *P* < 0.05; ^  (∗)^ *P* < 0.01; ^(‡)^ *P* < 0.001.*t*_1_: differences between column 1 and 2; *t*_2_: differences between column 3 and 4; *t*_3_: differences between column 1 and 3; *t*_4_: differences between column 2 and 4. POPQ: International Continence Society's Pelvic Organ Prolapse Quantification system. Aa: a point located in the midline of the anterior vaginal wall 3 cm proximal to the external urethral meatus; Ba: the most distal position of any part of the upper anterior wall from the vaginal cuff to point Aa; C: leading edge of the cervix; D: the depth of the Douglas recession (distance between the hymen and the most distal point of the Douglas); Bp: the most distal position of any part of the upper posterior wall from the vaginal cuff to point Ap; Ap: a point located in the midline of the posterior vaginal wall 3 cm proximal to the hymen; gh: genital hiatus, measured from the middle of the external urethral meatus to the posterior midline hymen; pb: perineal body, measured from the posterior margin of the genital hiatus to the midanal opening; tvl (total vaginal length): the greatest depth of the vagina in centimeters, that is, the distance between hymen and point D.

**Table 3:** Results of the Marshall coughing test during lithotomic position, lithotomic position with cervix reposition, and upright position in USIg (*n* = 132) and USIGPg(*n* = 136): USIGP(A) with stage I GP (*n* = 78) and USIGP(B) with stage II GP (*n* = 58).

Study groups	Marshall test in lithotomic position	Marshall test with CRM	Marshall test in upright position	Total (%)
USIg (*n* = 132)	(+)	(−)	(−)	0.0%
	(+)	(+)	(−)	0.0%
	(+)	(+)	(+)	10/132 (7.58%)
	(−)	(+)	(+)	0.0%
	(−)	(−)	(+)	20/132 (15.15%)
	(+)	(-)	(+)	102/132 (77.27%)

USIGP (A) with GP stage I (*n* = 78)	(+)	(−)	(−)	0.0%
	(+)	(+)	(−)	0.0%
	(+)	(+)	(+)	3/78 (3.85%)
	(−)	(+)	(+)	0.0%
	(−)	(−)	(+)	5/78 (6.41%)
	(+)	(−)	(+)	70/78 (89.74%) %)

USIGP (B) with GP stage II (*n* = 58)	(+)	(−)	(−)	8/58 (13.79%)
	(+)	(+)	(−)	0.0%
	(+)	(+)	(+)	0.0%
	(−)	(+)	(+)	0.0%
	(−)	(−)	(+)	20/58 (34.48%)
	(−)	(+)	(−)	30/58 (51.72%)

**Table 4:** Differences among the groups regarding the presence of USI during MT in all three positions.

(+) MT	USIg	USIGP(A)	USIGP(B)	*χ* ^2^		
	GP stage 0 (*n* = 132)	GP stage I(*n* = 78)	GP stage II (*n* = 58)			
	(1)	(2)	(3)	1-2	1–3	2-3
USI in lithotomic position	112/132 84.85%	73/78 93.60%	28/58 48.28%	0.69	**25.92** ^‡^	**38.34** ^‡^
USI with CRM	10/132	3/78	30/58	0.64	**49.91** ^‡^	**4.23** ^‡^
	7.57%	3.85%	51.72%			
USI in upright	132/132	78/78	20/68	0.00	**103.97** ^‡^	**67.79** ^‡^
	100.00%	100.00%	34.48%			

Mantel-Haenszel's *χ* ^2^test with df of 1: ^(‡)^ *P* < 0.001. POPQ: International Continence Society's Pelvic Organ Prolapse Quantification system. Stage 0: point Aa, Ba, Bp, and Ap at the level (−3), point C at the level (tvl-2); stage I: the most distal portion of the prolapse is >1 cm above the hymen; stage II: the most distal portion of the prolapse is less or equal to 1 cm proximal to or distal to the plane of the hymen.

**Table 5:** Differences among the groups regarding the division of patients and different combinations of the Marshall coughing test during the three positions.

Marshall test	USI	USIGP(A)	USIGP(B)	*χ* ^2^		
	(GP stage 0) (*n* = 132)	(GP stage I) (*n* = 78)	(GP stage II) (*n* = 58)			
	(1)	(2)	(3)	1-2	1–3	2-3
(+) in all three positions	10/132	3/78	0/58	0.62	**6.28** ^(†)^	0.85
	13.2%	5.17%	0.0%			
(+) only in up right position	20/132	5/78	0/58	1.48	**8.27** ^(∗)^	2.26
	15.15%	6.41%	0.0%			
(+) only in lithotomic position	0/132	0/78	8/58	0.00	**21.61** ^(‡)^	**14.07** ^(‡)^
	0.0%	0.0%	13.79%			
(−) only with CRM	102/132	70/78	20/58	**6.02** ^(†)^	**30.25** ^(‡)^	**43.00** ^(‡)^
	77.27%	89.74%	34.48%			
(+) only with CRM	0/132	0/78	30/58	0.00	**84.95** ^(‡)^	**101.74** ^(‡)^
	0.0%	0.0%	51.72%			

Mantel-Haenzel's *χ* ^2^ test with df of 1: ^(†)^ *P* < 0.05; ^  (∗)^ *P* < 0.01; ^(‡)^ *P* < 0.001.

**Table 6:** Functional symptoms according to the questionnaire and clinical examination in USIGPg (*n* = 136) with GP and USI, and USIg (*n* = 132) with USI.

	USIGPg (*n* = 136)	USIg (*n* = 132)	*χ* ^2^
	(1)	(2)
*Urinary symptoms (according to the questionnaire and clinical examination (Marshall test))*			
Potential USI (during prolapse reposition)	33/136 (24.3%)	10/132 (7.57%)	**12.61** ^(‡)^
Genuine USI (without prolapse reposition)	106/136 (77.8%)	132/132 (100.0%)	**34.98** ^(‡)^
Frequency	30/136 (22.1%)	20/132 (15.1%)	1.67 NS
Urgency	24/136 (19.6%)	29/132 (22.0%)	2.26 NS
Hesitancy	30/136 (22.1%)	25/132 (18.9%)	0.23 NS
Nocturia	19/136 (14.0%)	17/132 (12.9%)	0.03 NS
Incomplete emptying	49/136 (36.1%)	4/132 (3.0%)	**43.84** ^(‡)^
Weak stream	42/136 (30.9%)	4/132 (3.0%)	**30.66** ^(‡)^
*Bowel symptoms*			
Flatus incontinence	15/136 (11.0%)	0/132 (0.0%)	**13.38** ^(‡)^
Incontinence of liquid stool	4/136 (2.9%)	0/132 (0.0%)	2.19 NS
Urgency of defecation	19/136 (14.0%)	0/132 (0.0%)	**17.75** ^(‡)^
Discomfort with defecation	42/136 (30.9%)	4/132 (3.0%)	**30.66** ^(‡)^
Constipation	75/136 (55.1%)	25/132 (18.9%)	**35.94** ^(‡)^
Digital manipulation to finish defecation	19/136 (14.0%)	0/132 (0.0%)	**17.75 ** ^(‡)^
Feeling of incomplete evacuation	23/136 (16.9%)	4/132 (3.0%)	**12.73** ^(‡)^
Rectal protrusion during defecation	11/136 (8.9%)	0/132 (0.0%)	**9.15** ^(∗)^
*Sexual symptoms*			
Pain with coitus	109/136 (80.1%)	21/132 (15.9%)	**44.05 ** ^(‡)^
Unsatisfactory coitus	113/136 (83.1%)	12/132 (9.1%)	**144.14** ^(‡)^
Decrease in orgasmic response	117/136 (86.0%)	49/132 (37.1%)	**65.78 ** ^(‡)^
Incontinence during the intercourse	19/136 (14.0%)	45/132 (34.1%)	**16.01** ^(‡)^
*Other local symptoms*			
Vaginal pressure and heaviness	109/136 (80.1%)	0/132 (0.0%)	**174.68** ^(‡)^
Vaginal/perineal pain	34/136 (25.0%)	4/132 (3.0%)	**24.75** ^(‡)^
Low back pain	113/136 (83.1%)	4/132 (3.0%)	**170.97** ^(‡)^
Abdominal	106/136 (77.9%)	8/132 (6.1%)	**144.00** ^(‡)^

Mantel-Haenszel's *χ* ^2^ test with df of 1:  ^(†)^ *P* < 0.05; ^  (∗)^ *P* < 0.01; ^(‡)^ *P* < 0.001.

**Table 7:** Urodynamic analyses in USIGPg group (*n* = 136) with GP and USI, and USIg (*n* = 132) with USI.

Urodynamic parameters	USIGPg (*n* = 136)	USIg (*n* = 132)	*χ* ^2^
	(1)	(2)	1-2
*Cystometry*			
Detrusor instability	38/136 (27.9%)	33/132 (25.0%)	0.16
Decreased capacity of the bladder	57/136 (41.9%)	37/132 (28.0%)	5.06
Regular cystometry	98/136 (72.0%)	99/132 (75.0%)	0.47
*UPP max*	88.93±11,67	90.49 ± 14.22	0.25
*dUPP(default transmission)*			
Positive dUPP (USI (+) according to the urodynamic examination)	106/136 (77.9%)	124/132 (93.9%)	**15.40** ^(‡)^
Negative dUPP (USI (−) according to the urodynamic examination)	30/136 (21.1%)	8/132 (6.10%)	**12.78** ^(‡)^

Mantel-Haenszel's *χ* ^2^ test with df of 1:  ^(†)^ *P* < 0.05; ^  (∗)^ *P* < 0.01; ^(‡)^ *P* < 0.001.
